# Shaping the Future of Psychiatric Neurosurgery: From Connectomic Precision to Technological Integration

**DOI:** 10.3390/brainsci15060647

**Published:** 2025-06-16

**Authors:** Cristina V. Torres Díaz, Marta Navas García, Paloma Pulido Rivas, Mónica Lara Almunia, José Antonio Fernández Alén

**Affiliations:** Servicio de Neurocirugía, Hospital Universitario de La Princesa, 28006 Madrid, Spain; martasoti@yahoo.es (M.N.G.);

**Keywords:** psychiatric neurosurgery, deep brain stimulation, connectomics, artificial intelligence, obsessive–compulsive disorder, treatment-resistant depression, ethical neurotechnology, minimally invasive interventions, closed-loop systems, personalized medicine

## Abstract

Psychiatric neurosurgery is undergoing a profound transformation, propelled by advances in neurotechnology, connectomics, and personalized medicine. Once controversial, surgical interventions are now guided by detailed functional brain mapping and precise neuromodulation techniques, such as deep brain stimulation (DBS), which offer therapeutic options for patients with severe, treatment-resistant psychiatric disorders. This manuscript reviews the current techniques, including lesion-based procedures and DBS, and explores their mechanisms of action, from synaptic plasticity to large-scale network modulation. It highlights recent progress in neuroimaging, connectomic targeting, and artificial intelligence applications for surgical planning and the prediction of treatment responses. Ethical considerations—including informed consent, identity, and long-term follow-up—are critically examined in light of these advances. Furthermore, the growing role of minimally invasive procedures and wearable integrated neurotechnologies is discussed as part of a shift toward dynamic and adaptive interventions. Although still investigational, psychiatric neurosurgery is emerging as a technologically sophisticated field that demands rigorous clinical evaluation, ethical accountability, and an individualized approach to restoring function and autonomy in some of the most disabling mental illnesses.

## 1. Introduction

Psychiatric neurosurgery involves the application of surgical methods to alleviate severe mental illnesses that have not responded to conventional treatments. Unlike procedures used for neurological disorders such as epilepsy, Parkinson’s disease, or chronic pain—which focus primarily on improving motor or sensory function—psychiatric neurosurgery is aimed at modifying the brain circuits that regulate emotion, behavior, and cognition. This is typically achieved through highly targeted interventions, such as deep brain stimulation or the creation of small lesions in specific areas of the limbic system. Although the surgical techniques may resemble those used in other fields, psychiatric applications present distinct challenges, particularly with regard to patient selection, ethical safeguards, and the nuanced measurement of outcomes that affect personality, decision-making, and overall psychological well-being.

The trajectory of neurosurgery for psychiatric disorders has been marked by intense controversy and profound transformation. Since its origin in the early 20th century with prefrontal lobotomy—pioneered by António Egas Moniz in 1935 and later disseminated by Walter Freeman in the United States—the field has experienced a rapid rise and a precipitous fall. Freeman’s transorbital lobotomy, performed with minimal scientific rigor and indiscriminate selection, inflicted devastating consequences on thousands of patients and compromised the credibility of psychiatric surgery for decades [1,2].

The subsequent decades brought more refined approaches, such as anterior capsulotomy and cingulotomy, aimed at modulating the circuits involved in affective regulation and impulse control. Nonetheless, the emergence of effective pharmacological treatments in the 1950s and 1960s relegated psychosurgery to a marginal role [3].

Yet today, psychiatric disorders remain among the leading causes of disability worldwide. A significant proportion of patients with conditions such as major depression, obsessive–compulsive disorder, and bipolar disorder do not respond to pharmacological or psychotherapeutic interventions. In these treatment-resistant cases, symptoms persist despite optimal medical management, severely compromising quality of life, social functioning, and autonomy. This clinical reality underscores the urgent need for alternative and more effective therapeutic strategies—especially for those patients who have exhausted the conventional options.

In recent years, the field has re-emerged under a new paradigm. Lesional techniques, once regarded as crude interventions, have evolved into procedures of extraordinary precision. Technologies such as stereotactic radiofrequency, Gamma Knife radiosurgery, and high-intensity focused ultrasound (HIFU) now enable the creation of millimetric lesions in highly specific nuclei and fiber bundles, offering targeted structural neuromodulation with minimal collateral damage [4,5].

In parallel, deep brain stimulation (DBS) has gained prominence as a reversible and programmable modality. Originally developed for movement disorders, DBS has shown promising efficacy in conditions such as treatment-resistant obsessive–compulsive disorder, and is currently under investigation for major depression, Tourette’s syndrome, and other psychiatric conditions [3]. Its capacity to dynamically modulate neural networks represents a conceptual shift—from lesion-based ablation to circuit-level regulation [4].

This evolution has been propelled by advances in functional neuroimaging. Tools such as diffusion tensor imaging (DTI) and functional MRI (fMRI) now enable detailed mapping of the brain networks involved in emotion, cognition, and motivation [6]. Psychiatric surgery is no longer directed at isolated anatomical targets, but at dysfunctional neural circuits—an approach grounded in the understanding of mental illness as a disorder of large-scale, interconnected networks.

Looking ahead, the future of neurosurgery for psychiatric disorders is being reshaped by cutting-edge innovations. Adaptive stimulation systems (“closed-loop DBS”), artificial intelligence applied to surgical planning, and the development of non-invasive neuromodulation technologies are redefining therapeutic boundaries [7]. These advances, however, raise fundamental ethical questions: To what extent is it acceptable to intervene in brain circuitry to modulate emotional states? Can patients with impaired autonomy truly give valid informed consent?

This article explores the current status of, technological advances, and ethical challenges in psychiatric neurosurgery, offering a forward-looking perspective on its potential role in the treatment of severe, refractory mental illness. The references cited throughout this article were selected from the biomedical literature using PubMed as the primary source, without applying a restriction by year. The search terms included combinations such as “deep brain stimulation”, “psychiatric neurosurgery”, “treatment-resistant depression”, and “connectomic targeting”. While this is not a systematic review, the article aims to provide a concise synthesis of representative developments and forward-looking trends in the field.

## 2. Contemporary Clinical Practice in Psychiatric Neurosurgery

Contemporary psychiatric neurosurgery encompasses two primary modalities: lesion-based procedures and DBS. Both approaches aim to modulate the dysfunctional brain circuits implicated in severe mental disorders, but differ in their mechanisms, reversibility, and regulatory status.

Lesional techniques such as anterior capsulotomy and cingulotomy, historically performed with crude tools, are now executed with millimetric precision using stereotactic radiofrequency or radiosurgical devices. These interventions target structures like the anterior limb of the internal capsule or the anterior cingulate cortex, with the goal of disrupting pathological connectivity within fronto-striatal or limbic circuits. Gamma Knife radiosurgery, for example, allows for non-invasive lesioning without craniotomy, and has been used in selected cases of treatment-resistant obsessive–compulsive disorder (OCD), depression, and aggressive behavior syndromes [8]. HIFU, a more recent tool, offers the possibility of real-time, image-guided lesion creation with submillimetric accuracy and minimal collateral damage [9].

DBS, on the other hand, offers a non-lesional, adjustable, and reversible method of circuit modulation. Electrodes are surgically implanted into deep brain targets and connected to a programmable pulse generator, allowing for the chronic electrical stimulation of the neural structures implicated in emotional regulation, motivation, or cognitive flexibility. Initially developed for movement disorders like Parkinson’s disease and essential tremor, DBS has since been applied to psychiatric conditions, particularly OCD and major depressive disorder (MDD) [10]. Its efficacy in OCD is supported by randomized controlled trials, leading to conditional regulatory approval in several jurisdictions, although it is still considered an “emergent” and not a standard technique. Its use in depression remains investigational as well, with clinical trials yielding mixed results and raising important questions about target selection, patient stratification, and stimulation paradigms [11,12].

Despite advances, psychiatric neurosurgery remains reserved for the most severe, treatment-resistant cases. Multidisciplinary evaluation is essential, and strict selection criteria are enforced to ensure that surgical intervention is considered only after exhaustive pharmacological and psychotherapeutic strategies have failed. Psychiatric comorbidities and the patient’s insight, support system, and ability to adhere to long-term follow-up are also taken into account [13].

There is also an ongoing debate about optimal targets. While the anterior limb of the internal capsule and subgenual cingulate cortex have received considerable attention [14,15], other structures such as the nucleus accumbens, the medial forebrain bundle (MFB), the habenula, and the bed nucleus of the stria terminalis are being explored [16,17]. Functional neuroimaging and tractography are increasingly used to tailor target selection based on individual connectivity profiles—a shift toward personalized connectomic neurosurgery [18,19].

**Figure 1:**
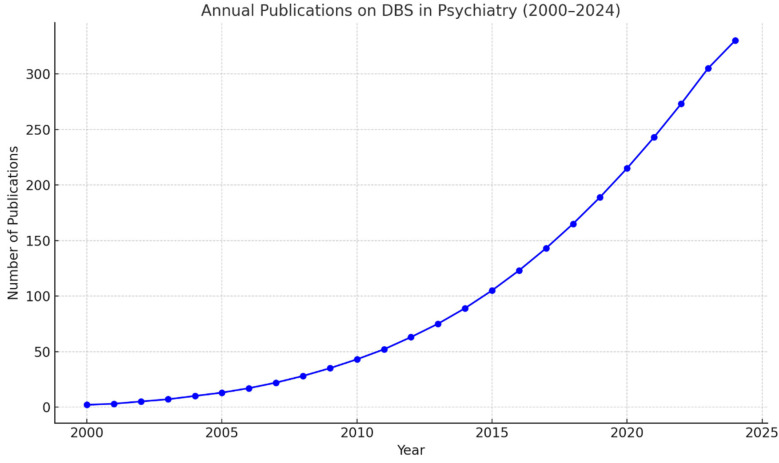
Annual scientific publications on DBS for psychiatric disorders (2000–2023). The figure illustrates the growing number of peer-reviewed articles indexed in PubMed related to DBS in psychiatry. The steady increase reflects the expanding scientific interest and development of neurosurgical strategies for treatment-resistant mental illnesses.

In parallel, efforts are underway to better understand the mechanisms of action of both DBS and lesional interventions, to define the most effective stimulation parameters and identify robust biomarkers of response. These investigations aim to clarify the neurobiological substrates of psychiatric disorders and to support the rational selection of surgical targets, ultimately improving clinical outcomes and patient selection (Figure 1).

## 3. Recent Neurobiological Insights and Implications for Neuromodulation

A more refined understanding of psychiatric pathophysiology is redefining therapeutic strategies and expanding the conceptual framework of DBS. While early models emphasized monoaminergic imbalance or isolated circuit dysfunction, recent research reveals more complex, systems-level alterations involving synaptic plasticity, immune signaling, gut–brain interactions, and large-scale network connectivity.

In major depression, classical theories have long focused on serotonin, noradrenaline, and dopamine deficiencies to explain affective and motivational symptoms. While these models supported the rationale for pharmacological interventions, emerging evidence shows that depression is also characterized by disrupted synaptic homeostasis, neuroinflammation, and the dysfunction of the blood–brain barrier [20,21]. Such findings shift the therapeutic perspective beyond neurotransmitter modulation and suggest the potential for DBS to target not only specific circuits, but also network-level and neuroimmune dysregulation.

Similarly, in OCD, classical circuit-based models have centered on hyperactivity in the cortico-striato-thalamo-cortical loop, particularly involving the orbitofrontal cortex and basal ganglia. However, newer data highlight the role of the gut–brain axis and endocannabinoid signaling in OCD pathophysiology. The transplantation of microbiota from OCD patients into mice induces compulsive-like behaviors and neuroinflammatory changes, and alterations in cannabinoid gene expression have been identified in human patients [22,23]. These findings open up new avenues for understanding OCD as a multisystem disorder and refining DBS targets accordingly.

Anorexia nervosa, traditionally linked to altered serotonergic and dopaminergic signaling, is now also associated with accelerated brain aging and impaired reward learning. Neuroimaging shows reduced gray matter volumes during the acute phase and persistent cognitive inflexibility, even after weight restoration [24,25]. Such insights support the investigation of DBS interventions targeting reward and cognitive control circuits to address treatment-resistant forms of the disorder.

In schizophrenia, the longstanding dopaminergic hypothesis has been enriched by connectomic models that describe the disorder as one of large-scale dysconnectivity, particularly involving frontotemporal and salience networks. Research now implicates neuroinflammation, NMDA receptor hypofunction, and gut microbiota disturbances in disease onset and progression [26,27]. These findings reinforce the rationale for neuromodulatory approaches that go beyond symptom suppression to circuit-level correction and immune modulation.

These conditions—depression, OCD, anorexia, and schizophrenia—are presented here as illustrative examples. Other disorders, such as treatment-refractory aggression and Gilles de la Tourette syndrome, also exhibit neurobiological abnormalities that may be amenable to neurosurgical intervention. As the field advances, DBS is increasingly being conceptualized not simply as a symptomatic intervention, but as a strategy grounded in emerging models of brain circuit dysfunction and systemic interaction.

## 4. Mechanisms of Action: Emerging Insights into DBS for Psychiatric Disorders

DBS has emerged as a promising intervention for treatment-resistant psychiatric disorders. In clinical practice, DBS is not a first-line treatment for psychiatric conditions, but rather a highly specialized option reserved for patients with severe, treatment-resistant forms of illness. For instance, in MDD, DBS is typically considered only after the failure of multiple pharmacological treatments and at least one adequate trial of electroconvulsive therapy (ECT). Even then, it is indicated only for a carefully selected subgroup of patients with chronic, debilitating symptoms that significantly impair functioning. Similarly, in OCD, DBS is indicated for patients with severe and enduring symptoms that persist despite optimized pharmacotherapy with serotonin reuptake inhibitors and augmentation with antipsychotics, as well as cognitive behavioral therapy based on exposure and response prevention. These stringent criteria ensure that DBS is reserved for cases with the greatest potential benefit and the least likelihood of spontaneous recovery or response to less invasive therapies.

Recent research has elucidated its multifaceted mechanisms of action, encompassing neurophysiological modulation, network-level adjustments, and molecular alterations.

At the cellular level, high-frequency DBS modulates neuronal activity by inducing both inhibitory and excitatory effects, disrupting the pathological neural oscillations associated with psychiatric symptoms. For instance, elevated theta rhythms in the subgenual cingulate cortex correlate with negative emotional states; DBS targeting this region can attenuate such aberrant activity, leading to symptom improvement [28]. These effects are not confined to the stimulation site, but extend to broader neural circuits, restoring the functional connectivity between cortical and limbic regions [29].

DBS also influences the large-scale brain networks implicated in psychiatric conditions. The dysregulation of the default mode network (DMN), central executive network (CEN), and salience network (SN) contributes to symptoms such as rumination and impaired cognitive control. DBS has been shown to recalibrate these networks, decreasing hyperactivity in the DMN and enhancing CEN function, thereby improving emotional regulation and cognitive flexibility [30].

On a molecular level, DBS induces neurochemical changes that support its therapeutic effects. It can increase the release of neurotransmitters like dopamine, serotonin, and norepinephrine in targeted brain regions, counteracting the deficits observed in various psychiatric disorders [31]. Moreover, DBS promotes synaptic plasticity by upregulating neurotrophic factors such as brain-derived neurotrophic factor (BDNF), facilitating neuronal resilience and network reorganization [32].

These insights underscore the complex, multi-level impact of DBS on the brain, highlighting its potential as a versatile tool in the treatment of refractory psychiatric conditions. Ongoing research continues to refine our understanding of these mechanisms, aiming to optimize DBS protocols and expand its applicability across diverse psychiatric populations.

## 5. Cutting-Edge Technologies Driving the Evolution of Psychiatric Neurosurgery

Recent decades have witnessed a paradigm shift in psychiatric neurosurgery, catalyzed by the convergence of neuroscience, engineering, and computational modeling. The field is rapidly moving from static, lesion-based approaches to dynamic, personalized, and network-guided interventions. The following technological advances are reshaping the way we conceptualize and implement surgical treatments for psychiatric disorders.

### 5.1. Real-Time Responsiveness in DBS: The Emergence of Closed-Loop Systems

A major innovation in psychiatric neurosurgery lies in the development of adaptive or closed-loop DBS systems. These devices mark a departure from conventional continuous (open-loop) stimulation by offering an intelligent, feedback-driven alternative. Rather than operating with fixed parameters, adaptive DBS systems monitor neural activity and respond selectively when pathological brain states are detected, aligning stimulation with the real-time needs of the patient.

This dynamic approach has proven particularly beneficial in disorders marked by fluctuating symptoms, such as depression and OCD. For instance, Scangos et al. designed a closed-loop protocol in which gamma oscillations in the amygdala served as a real-time biomarker to trigger the stimulation of the ventral capsule/ventral striatum. The result was a rapid and sustained remission in a patient with severe, treatment-resistant depression, with stimulation delivered only during episodes of heightened neural activity [33].

In OCD, Widge et al. [19] leveraged frontal theta activity as a trigger for DBS. Their system delivered stimulation only when the orbitofrontal theta power—linked to obsessive thoughts—exceeded a certain threshold, allowing for more precise and efficient symptom control without overexposure to stimulation [19].

Beyond these individual cases, broader clinical studies are helping to establish the reliability and generalizability of this strategy. For example, Provenza et al. explored closed-loop stimulation in both OCD and MDD using the ventral capsule/ventral striatum as a target, and demonstrated that changes in frontal theta oscillations reliably tracked symptom-related cognitive states [34]. These findings reinforce the idea that effective neuromodulation hinges not just on target selection, but on timing—adapting stimulation to moments of maximum pathological activity.

In parallel, computational tools are increasingly being integrated into closed-loop architectures. Machine learning algorithms can detect subtle neural patterns predictive of symptom onset, enabling DBS devices to “learn” and refine their response strategies over time. For example, adaptive control algorithms have been trained to modulate stimulation in response to mood-related signals detected via intracranial EEG, offering a glimpse into the future of highly personalized, AI-driven brain–computer interfaces [35].

Despite these advances, challenges remain. The field continues to explore how best to define reliable biomarkers across heterogeneous psychiatric presentations, and how to design systems that remain safe, efficient, and user-friendly in real-world settings. Nonetheless, the movement toward adaptive DBS represents a conceptual shift: treating mental illness as a state-dependent neural dysfunction and delivering intervention with temporal precision.

### 5.2. Precision Targeting Through Personalized Connectomic Neurosurgery

The traditional approach to psychiatric surgery relied heavily on standardized anatomical atlases—assuming that certain targets, such as the anterior limb of the internal capsule or subthalamic structures, would yield consistent results across patients with similar diagnoses. Yet, clinical experience has shown that even when electrodes are placed in the same location, outcomes can vary widely. This discrepancy has fueled a conceptual shift: the recognition that psychiatric symptoms arise not from isolated brain regions, but from dysfunctional communication between distributed circuits. As such, modern neurosurgical planning is increasingly guided by each patient’s unique connectivity profile, moving beyond fixed coordinates toward network-informed targeting.

Advances in neuroimaging, particularly diffusion tensor imaging (DTI) and resting-state functional MRI (rs-fMRI), have enabled the in vivo reconstruction of white matter tracts and intrinsic functional connectivity. These techniques allow clinicians to visualize how potential stimulation sites are embedded within broader circuits, identifying the pathways most likely to mediate clinical improvement. In this framework, the efficacy of DBS depends not solely on anatomical placement, but on the ability of an electrode to engage the symptom-relevant networks.

Horn and Fox were among the first to empirically validate this approach. By reanalyzing data from over 100 psychiatric patients treated with DBS, they demonstrated that the degree of connectivity between the electrode site and the ventromedial prefrontal cortex (vmPFC) was a robust predictor of therapeutic outcomes. Strikingly, this single metric accounted for nearly half of the variance in treatment responses—underscoring the primacy of functional network modulation over anatomical precision [36].

Subsequent studies have reinforced this principle. In a prospective trial, Li et al. [37] applied individualized tractography-based targeting in 24 OCD patients. By mapping each patient’s structural connectivity, surgical planning was optimized to direct stimulation toward fibers projecting to the medial prefrontal cortex. This personalized approach led to clinically significant improvements in 70% of cases, demonstrating that circuit-level targeting is not just theoretically appealing, but also clinically effective [37].

Baldermann et al. [38] extended these findings by identifying a specific fiber tract connecting the anterior internal capsule to the dorsomedial prefrontal cortex, whose activation correlated with dramatic symptom relief. In their OCD cohort, targeting this tract produced a 60% average reduction on the Y-BOCS scale—among the most impressive results ever reported in neurosurgical psychiatry [38].

The predictive power of connectivity has also been demonstrated using machine learning. Al-Fatly et al. combined high-resolution tractography with computational models in a cohort of 245 DBS patients. Their method generated individualized “connectomic fingerprints” that predicted treatment response with over 80% accuracy, marking a shift toward neuroanatomical precision medicine [39].

Together, these studies provide compelling evidence to suggest that functional network engagement, not mere anatomical placement, determines DBS efficacy. Surgical planning informed by connectomics offers a more precise, biologically grounded path to optimizing psychiatric outcomes, while also deepening our understanding of how stimulation alters brain function. This personalized approach—rooted in each patient’s real-time neuroarchitecture—has the potential to redefine psychiatric neurosurgery in the coming decade.

### 5.3. Artificial Intelligence Applied to Personalized Surgery

The growing integration of artificial intelligence (AI) into medicine is beginning to revolutionize the field of functional neurosurgery for psychiatric disorders. In contrast to traditional approaches that rely on broad clinical criteria and expert intuition, AI-driven tools offer the potential to predict treatment responses, tailor surgical planning, and adjust stimulation parameters with unprecedented precision.

One of the earliest breakthroughs in this area was achieved by Pulini et al. [40], who developed supervised machine learning models to classify patients based on neurofunctional biomarkers extracted from EEG and fMRI data. Their algorithms successfully identified clinically relevant signal patterns, offering a robust method for stratifying candidates for neuromodulation based on individualized brain signatures [40]. These tools provide a crucial link between the rich complexity of neuroimaging data and its translation into clinically actionable insights.

Further evidence comes from the work of Habets et al. [41], who applied machine learning to a cohort of 32 patients undergoing DBS of the subgenual cingulate cortex for treatment-resistant depression. By integrating neuroanatomical, functional, and clinical variables, their models predicted responses to DBS with an impressive 87% accuracy—highlighting the potential of algorithmic forecasting in guiding surgical decisions even before intervention takes place. Although the study by Habets et al. reported impressive predictive accuracy in using machine learning to forecast DBS responses in OCD patients, it is important to interpret these results with caution due to the limited sample size, which may restrict generalizability and requires validation in larger, independent datasets [41]. This capacity to anticipate outcomes may carry significant ethical and economic implications, facilitating more targeted, evidence-based care.

Artificial intelligence is also reshaping how stimulation is delivered. In an adaptive DBS context, Widge et al. [19] embedded deep neural networks into closed-loop systems capable of modulating stimulation in response to real-time neural states. These systems learned to detect early signs of pathophysiological transitions—such as the onset of an obsessive or depressive episode—and adjusted stimulation accordingly, without human input [19]. This development transforms the DBS device into an autonomous neurocognitive interface: a system capable of decoding, interpreting, and correcting pathological brain activity as it occurs.

Beyond individual cases, AI has also been applied at scale. Gadot et al. [42] trained deep learning models using multimodal datasets—including connectomic imaging, clinical data, and neuropsychological measures—from multicenter cohorts of OCD patients. Their models achieved over 80% accuracy in predicting DBS outcomes, demonstrating that scalable predictive tools can be developed from diverse and heterogeneous populations [42]. This approach lays the groundwork for generalizable decision-support systems that move beyond anecdotal or case-by-case insights.

Altogether, these findings suggest that AI is not merely an add-on to surgical psychiatry, but a fundamental force reshaping its logic and methods. By enabling real-time adaptation, precise patient stratification, and large-scale predictive modeling, artificial intelligence is driving a shift toward neurocomputational psychiatry—a paradigm in which mental disorders are understood and treated through the interplay of circuits, data, and dynamic modeling. The future of psychiatric neurosurgery will likely be defined by this convergence of biology, computation, and technology.

### 5.4. Minimally Invasive Strategies in Psychiatric Neurosurgery: From Energy-Based Lesions to Biological Interfaces

In recent years, the refinement of minimally invasive neurosurgical procedures has expanded the treatment landscape for psychiatric disorders, particularly for patients with severe and treatment-resistant symptoms who are ineligible or unwilling to undergo more invasive interventions like DBS. These approaches seek to modulate pathological brain circuits without craniotomy or permanent implants, thereby reducing surgical risk, improving recovery times, and enhancing acceptability from both a clinical and patient perspective. Still, when applied to deep brain structures or in the absence of standardized protocols, they may carry neurological or cognitive risks that warrant thorough investigation.

Among these techniques, HIFU has gained attention for its ability to create precise thermal lesions in targeted areas of the brain using converging ultrasound beams, guided in real-time by MRI. In the treatment of OCD, this method has been used to perform bilateral capsulotomies of the anterior limb of the internal capsule (ALIC), a classical neurosurgical target due to its dense connectivity with the prefrontal and subcortical circuits involved in compulsivity and intrusive thoughts.

A pilot study by Jung et al. [5] demonstrated the feasibility of HIFU in this context by treating four patients with refractory OCD. Bilateral capsulotomies were carried out under MR guidance, and three of the four patients achieved a clinically significant improvement, defined as a ≥35% reduction in Y-BOCS scores. The intervention was generally well tolerated; no patients demonstrated any side effects (physical or neuropsychological) in relation to the procedure. In addition, there were no significant differences found in the comprehensive neuropsychological test scores between the baseline and 6-month time points [5].

These promising findings were extended in a larger prospective series conducted by Kim et al. [43], involving 11 patients who underwent the same HIFU procedure. After a two-year follow-up period, the average Y-BOCS score decreased from 34.4 to 21.3, reflecting a substantial reduction in obsessive–compulsive symptoms. In parallel, comorbid depressive and anxious symptomatology also improved markedly, with HAM-D scores falling from 19.0 to 7.6 and HAM-A scores from 22.4 to 7.9. Global functioning, measured by the GAF scale, improved from an average of 35.8 to 56.0. Six patients (54.6%) were classified as full responders, two (18.2%) as partial responders, and one (9.1%) achieved full remission. Adverse effects were generally mild and transient, with headache (63.6%), vestibular symptoms such as nausea or dizziness (45.5%), and transient anxiety (27.3%) being the most commonly reported. No serious neurological events or significant cognitive impairments were observed [43].

The long-term efficacy of MR-guided focused ultrasound (MRgFUS) capsulotomy was evaluated in a 10-year follow-up study, which further supported the durability of this approach. In that cohort, 70% of patients met the criteria for full response, with two individuals achieving full clinical remission (Y-BOCS ≤ 12 and CGI-S = 1 or 2). Symptom severity continued to decline over time—Y-BOCS scores decreased from 20.7 after two years to 16.4 after ten years (*p* = 0.012)—and global functioning improved from 57.4 to 69.0 on the GAF scale (*p* = 0.011). Frontal lobe-related cognitive functions also showed improvement, and no cases of suicide, long-term neurological deficits, or serious complications were reported. Patient satisfaction with the treatment was high across the sample [44].

Gamma Knife capsulotomy (GKC) represents another minimally invasive stereotactic technique that has been used for decades in the neurosurgical management of OCD. This method relies on multiple converging beams of gamma radiation to produce localized lesions without the need for incision, anesthesia, or implanted devices. In a clinical series by Lopes et al. [5], 16 patients with severe, refractory OCD underwent bilateral capsulotomies targeting the ventral portion of the ALIC. Sustained symptomatic relief, defined as a ≥35% improvement on the Y-BOCS scale, was achieved in 56% of cases. Some patients presented with mild, reversible executive dysfunction, likely related to the incidental involvement of neighboring frontostriatal fibers; however, its overall safety profile and efficacy supported its use as a second-line option in appropriately selected individuals [5].

Recent mechanistic insights into GKC have been provided by Jean Régis et al. [45], who introduced the concept of radiomodulation to describe the non-lesional effects of low-dose focused radiation on brain circuits. Their research suggests that even subnecrotic doses can produce significant functional modulation of neural activity—especially within the ALIC—by altering the axonal excitability, connectivity patterns, and glial signaling before any detectable structural damage occurs. These effects were initially observed in patients undergoing radiosurgical treatment for epilepsy and have since been extended to psychiatric contexts such as OCD. In this framework, therapeutic benefit may result not only from ablation, but also from radiation-induced plasticity, including white matter reorganization and circuit-level rebalancing [45].

At the experimental end of the spectrum lies irreversible electroporation (IRE), a novel neurosurgical technique that applies brief, high-intensity electric pulses to open pores in cell membranes, resulting in apoptotic cell death while preserving the extracellular matrix, vasculature, and myelin. This biophysical mechanism makes IRE particularly attractive for lesioning within delicate or highly vascular brain regions, such as the limbic system. Although it has not yet been used in psychiatry, early studies in animal models suggest that it can offer the focal modulation of target circuits with minimal collateral damage [46].

Altogether, these minimally invasive technologies represent an evolving frontier in the surgical treatment of psychiatric illness. Their shared goal is to maximize network-specific therapeutic modulation while reducing invasiveness and surgical risk. Nonetheless, their clinical implementation must be accompanied by rigorous assessment, standardized outcome metrics, and careful ethical oversight as the field moves toward broader adoption.

## 6. Expanding Targets and Networks: A New Paradigm in Psychiatric Neurosurgery

Recent advances in psychiatric neurosurgery extend far beyond technological innovation. A profound conceptual shift is underway: surgical targeting is no longer guided solely by fixed anatomical coordinates, but increasingly informed by our growing understanding of the brain’s dynamic functional architecture. Using tools such as functional MRI and tractography, neurosurgeons are now able to plan interventions that engage specific pathological circuits—opening the door to more precise modulation, better clinical outcomes, and fewer side effects. This evolution not only enhances efficacy, but also raises important scientific and ethical questions regarding the scope and personalization of neurosurgical interventions.

### 6.1. The Medial Forebrain Bundle: Reigniting the Reward Circuit

One of the most promising emerging targets in this landscape is the medial forebrain bundle (MFB). Although a relatively compact structure, it plays a pivotal role in regulating reward processing, motivation, and positive affect. The MFB serves as a key conduit in the mesolimbic dopaminergic pathway, linking the ventral tegmental area (VTA) with the nucleus accumbens, medial prefrontal cortex, and orbitofrontal cortex—regions critical in goal-directed behavior and hedonic capacity [47,48].

Anatomically, the MFB provides a rapid and direct route for dopaminergic signaling, modulating the cortical activity linked to emotional evaluation, initiative, and subjective well-being. In treatment-resistant depression, hypofunction of this mesolimbic system has been consistently observed, particularly in relation to core symptoms like anhedonia, apathy, and existential despair—symptoms notoriously resistant to pharmacotherapy [47,48].

Schlaepfer et al. pioneered clinical applications of MFB targeting. In their phase I pilot trial, seven patients with refractory major depression received unilateral stimulation of the superolateral branch of the MFB. Response rates reached 85%, with 57% of patients achieving full remission after one year—outcomes among the most robust reported in surgical psychiatry [47,48]. Strikingly, some patients reported marked clinical improvements within just 48–72 h post implantation, suggesting that stimulation of the MFB can produce the rapid modulation of reward circuits even before structural cortical reorganization occurs.

This accelerated onset contrasts with the delayed effects typically seen in subgenual or accumbens-targeted DBS. Further studies, such as those by Fenoy et al., confirmed these effects, highlighting improvements in motivational drive, hedonic tone, and affective reactivity—symptom clusters closely associated with poor prognosis when left untreated [49].

Tractography-based navigation has been key to safely targeting the MFB, allowing surgeons to avoid neighboring critical structures such as the lateral hypothalamus or thalamocortical fibers. Importantly, no serious neuropsychiatric complications have been reported when stimulation has been guided with anatomical precision and followed with close clinical monitoring. The MFB thus represents not just a new target, but a strategic shift in focus—from top-down control to the bottom-up restoration of motivation.

### 6.2. The Orbitofrontal Cortex: Modulating Impulse and Affective Valuation

The orbitofrontal cortex (OFC) has become another target of interest, given its crucial role in affective decision-making, reward valuation, and behavioral inhibition. Functionally positioned at the intersection of limbic, striatal, and prefrontal networks, the OFC is essential for evaluating emotional significance, predicting outcomes, and flexibly adjusting behavior.

Neuroimaging has repeatedly shown medial OFC hyperactivity in disorders such as OCD, where it contributes to persistent rumination, cognitive rigidity, and compulsive behavior. It is also implicated in impulsive dyscontrol, seen in addiction and certain personality disorders [50].

In this context, Figee et al. demonstrated that DBS of the medial OFC significantly reduced obsessive symptoms and enhanced cognitive flexibility in patients with refractory OCD [51]. The stimulation of this region appeared to normalize the aberrant activity in orbitofronto-striatal loops, improving patients’ capacity to disengage from intrusive thoughts and adapt to changing environmental demands.

Beyond OCD, studies in substance use disorders suggest that OFC modulation can attenuate craving and reduce reactivity to drug-associated cues—mechanisms central to relapse vulnerability. These findings position the OFC as a promising node for interventions targeting both affective control and impulse regulation [52].

### 6.3. The Lateral Habenula: Alleviating Despair at the Source

The lateral habenular nucleus (LHb), though small, has emerged as a compelling target due to its inhibitory role over the dopaminergic midbrain. It serves as a relay for aversive processing and is activated in response to negative prediction errors. Sustained LHb hyperactivity correlates with persistent dopaminergic suppression—a neurobiological correlate of hopelessness and anhedonia [53,54,55,56].

The first clinical application of LHb DBS was reported by Sartorius et al. in a patient with severe TRD who achieved stable remission following implantation [54]. Subsequent studies, including the work of Zhang et al. [55], extended this approach to a broader cohort. In their open-label pilot trial, seven patients with TRD or bipolar depression underwent bilateral LHb DBS. At one month, depression and anxiety scores had declined by nearly 50%, with consistent improvement in functioning and quality of life. These gains were sustained over 12 months, with average symptom reductions reaching 64% for depression and 70% for anxiety [55].

Neurophysiologically, local field potential recordings showed that patients with less synchronized oscillatory activity in the LHb had more severe symptoms at baseline, suggesting a link between habenular rhythm and clinical state. While some adverse events were observed—such as a transient hypomanic state in two patients and one case of acute mania requiring hospitalization—the overall safety profile was acceptable under close monitoring.

A recent systematic review confirmed that multiple clinical trials are currently investigating LHb DBS for mood disorders, with early findings indicating substantial therapeutic potential [56]. However, most studies emphasize the need for controlled trials to establish efficacy, safety, and optimal stimulation parameters [56,57,58].

Taken together, these novel targets—MFB, OFC, and LHb—represent the forefront of a conceptual revolution in psychiatric surgery. Rather than intervening in broad anatomical regions, modern neurosurgery now aims to recalibrate the dysfunctional networks tailored to specific symptom domains such as anhedonia, cognitive rigidity, or affective instability. This shift brings the field closer to truly personalized brain modulation.

## 7. Toward Connectomic Precision: Tailoring Surgery to Individual Brain Architectures

A paradigm-defining advancement in psychiatric neurosurgery is the move toward fully individualized interventions, guided by each patient’s specific brain connectivity profile. This shift acknowledges that identical psychiatric diagnoses may arise from fundamentally distinct neuroanatomical configurations, and that successful outcomes depend less on targeting a fixed anatomical point and more on modulating the functional connections between deep structures and cortical networks [37].

With the integration of tools such as DTI and resting-state functional MRI, it is now possible to trace the axonal pathways that connect key subcortical targets in vivo—like the subgenual cingulate, internal capsule, or nucleus accumbens—to areas of the prefrontal cortex involved in mood, decision-making, and motivation. These personalized connectivity maps are increasingly used to refine electrode placement or focus lesioning procedures for maximum therapeutic precision [37].

An illustrative case of this strategy is the work of Li et al. [37], who introduced a tractography-based planning method for DBS in treatment-resistant OCD. By identifying the fiber pathways with optimal connectivity to the medial prefrontal cortex in each subject, they achieved a significant clinical response in 70% of their participants—a compelling demonstration of the superiority of connectivity-based over purely anatomical targeting [37].

In a complementary development, Mosley et al. [59] created an AI-driven framework for predicting DBS outcomes by combining structural connectivity metrics, functional brain data, and machine learning. Their models enable preoperative simulations of therapeutic responses and assist in selecting optimal stimulation trajectories while minimizing adverse effects. This represents a major step toward predictive, precision-guided, psychiatric neurosurgery [59].

Together, these innovations mark the end of the “universal target” era and inaugurate a new model of flexible, patient-specific intervention—one grounded in empirical connectomic data. With these advances, psychiatric neurosurgery is evolving into a truly adaptive field, capable of responding to the intricacies of each individual’s brain and providing more effective, ethically sound, and safer treatment strategies.

## 8. Ethical Challenges in Psychiatric Neurosurgery: Consent, Identity, and Authenticity

Psychiatric neurosurgery has undergone a profound transformation since its controversial origins in the 20th century. Contemporary procedures are no longer defined by the crude and indiscriminate techniques of the lobotomy era, but by a refined understanding of the neural circuits implicated in mental illness and the use of highly targeted neuromodulation technologies. Yet, as the field advances in efficacy and precision, it also faces increasingly complex ethical challenges that compel a reexamination of the boundaries of therapeutic intervention in the human brain.

Unlike neurosurgical treatment for neurological conditions such as Parkinson’s disease or epilepsy—which primarily aim to alleviate motor or sensory symptoms—interventions in psychiatry may directly affect aspects fundamental to personhood, including emotion, volition, moral judgment, and identity. This raises critical ethical questions: Is it acceptable to alter the brain circuits associated with personality or affect? And how can we ensure that consent is truly informed and voluntary when the very disorders being treated may impair insight, decision-making, or motivation?

Moreover, psychiatric surgery must contend with persistent risks of stigma, unequal access, and the potential for premature clinical use driven by commercial or institutional pressures rather than robust evidence. Navigating these concerns will be essential to ensure that the future of psychiatric neurosurgery is grounded not only in scientific rigor, but also in ethical responsibility and equitable care for those affected by the most severe psychiatric conditions.

### 8.1. Informed Consent and Decisional Capacity in Psychiatric Neurosurgery

Ethical considerations in psychiatric neurosurgery extend far beyond the technical domain. At the forefront lies the issue of informed consent—an essential foundation of any medical intervention, but one that acquires unique complexity in the psychiatric context. For a patient’s consent to be valid, it must reflect an adequate understanding of the procedure, a rational evaluation of the risks and benefits, and the autonomous expression of a decision. Yet, many candidates for psychiatric surgery—such as those suffering from treatment-resistant depression, severe OCD, or profound anhedonia—present cognitive and emotional impairments that can compromise these very faculties.

Deficits have been documented in the domains essential for informed decision-making: the comprehension of medical information, the ability to weigh alternatives logically, the appreciation of one’s own condition, and the capacity to express consistent choices over time. In fact, studies estimate that 30–40% of individuals with severe psychiatric disorders show impairments in at least one of these areas, with even higher rates during active psychosis. This does not imply that all psychiatric patients are incapable of consenting, but rather that their capacity must be carefully and individually assessed. Clinical tools like the MacArthur Competence Assessment Tool for Treatment (MacCAT-T) provide a systematic framework to evaluate decision-making competence across the key domains of understanding, reasoning, appreciation, and expression of choice [60,61].

Furthermore, consent in this setting must be considered an ongoing process rather than a one-time event. Patients should receive progressive, comprehensible information, opportunities for reflection and consultation with trusted individuals, and repeated chances to confirm or revise their decisions. The participation of clinical ethics committees, capacity assessments by independent professionals, and access to second opinions reinforce this safeguard. It is also essential to acknowledge that many psychiatric neurosurgical interventions remain investigational, with benefits not yet guaranteed—an aspect that must be communicated transparently to protect patient autonomy [60,61].

### 8.2. Identity, Authenticity, and the Self: Ethical Reflections on Personality and Change

Closely tied to the ethics of consent is another central concern: the potential impact of neurosurgery on personal identity. Patients, families, and even clinicians sometimes worry that modulating circuits linked to emotion, judgment, or social behavior—such as the subgenual cingulate, nucleus accumbens, or orbitofrontal cortex—might fundamentally alter who the patient is. While such concerns are understandable, decades of clinical experience suggest that transformative personality changes are rare. Rather than creating a new self, these procedures tend to alleviate the pathological patterns that obscure the patient’s authentic identity.

Many individuals with severe psychiatric conditions describe feeling hijacked by symptoms that override their intentions, values, and preferences. When these symptoms remit following surgery, what re-emerges is not an altered identity, but a more liberated and functional version of the self. Patients frequently express this transformation with phrases like “I feel like myself again” or “now I can choose without OCD deciding for me” [3,7,9]. In this view, surgery facilitates a restoration of autonomy rather than a substitution of personality.

This process can sometimes lead to shifts within the family system, particularly after long periods of dependence. When the patient regains independence, caregivers may experience a sense of role displacement or uncertainty—a dynamic known as “displaced caregiver syndrome”. Far from being pathological, this adjustment phase is part of the reintegration process. Anticipating and supporting it through psychotherapeutic guidance can ease the transition for both the patient and their environment [62].

## 9. Scientific Rigor and Ethical Frameworks in the Advancement of Psychiatric Neurosurgery

The evolution of functional neurosurgery for psychiatric disorders—particularly DBS—must be firmly grounded in both robust clinical evidence and established international ethical standards.

The Declaration of Helsinki emphasizes that any experimental medical intervention must be supported by well-structured scientific research and approved by independent ethics committees. It also recognizes the possibility of compassionate use in exceptional circumstances—when patients face severe, treatment-refractory disorders, no effective standard therapies exist, the proposed intervention is scientifically plausible, or the decision is ethically reviewed and based on a fully informed consent process [63].

Additional international guidelines, such as the CIOMS Ethical Guidelines [64] and the European Regulation 536/2014 on clinical trials [65], further reinforce these principles, ensuring that clinical research balances individual protection with broader societal benefit.

In the specific case of DBS for treatment-resistant major depression or Gilles de la Tourette syndrome, clinical trial design has encountered unique challenges. One is the microlesional or insertion effect, whereby temporary symptom relief occurs from electrode implantation alone, regardless of active stimulation. Another is the delayed onset of therapeutic responses, which complicates early efficacy assessment and blurs the distinction between active and sham interventions.

These obstacles were highlighted in two major multicenter trials. The first, led by Mayberg et al., tested DBS targeting the subgenual cingulate using a randomized design, but was discontinued due to a lack of significant differences at the 6-week mark, despite showing sustained improvements during subsequent phases [28]. Similarly, Holtzheimer et al. conducted a double-blind trial that did not reach statistical significance after 16 weeks, though many participants exhibited meaningful clinical improvement in the open-label extension [66]. These findings illustrate that the design of psychiatric trials is just as critical as the intervention itself.

Conversely, the results in OCD have been more consistently positive. In an open-label study with one year of follow-up, Denys et al. demonstrated that 50% of patients with refractory OCD who received DBS targeting the nucleus accumbens experienced a reduction of ≥35% in their Y-BOCS scores [67]. Similarly, Mallet et al. conducted a double-blind crossover trial in which DBS of the subthalamic nucleus yielded significantly greater symptom reduction during the active stimulation phase compared to the sham phase [13].

At present, more than 50 clinical trials investigating DBS for psychiatric conditions are registered internationally. Among them are the following:

- TRANSCEND, a controlled crossover study exploring DBS for treatment-resistant depression with longitudinal follow-up [68].
- FORESEE III, a randomized trial assessing stimulation of the superolateral medial forebrain bundle, with targeting guided by individualized connectomic analysis [69].

While compassionate use may be justified in select cases, the preferred path for advancing psychiatric neurosurgery remains through rigorous clinical research. This includes innovative methodologies like single-case (N = 1) trials, which enable detailed monitoring, systematic data collection, and meaningful contributions to the growing scientific literature.

## 10. Integration of Surgery and Wearable Neurotechnology

An emerging frontier in psychiatric neurosurgery is the integration of surgical interventions with wearable neurotechnology—an evolution that could transform how implanted therapies are monitored, adjusted, and optimized over time. This convergence is made possible by simultaneous advances in three key areas: implantable stimulation systems with real-time neural recording capabilities, portable tools for continuous cognitive and behavioral assessment, and intelligent algorithms capable of adapting therapy based on environmental and behavioral data.

In the case of DBS, next-generation devices are being equipped with the ability to capture LFPs and other neural signals, enabling adaptive closed-loop stimulation that responds dynamically to fluctuations in the patient’s brain state [70]. These systems are increasingly designed to interface with external wearable devices—such as smartwatches, sleep monitors, speech analysis tools, facial affect detection software, and app-based self-report platforms—facilitating the creation of real-time, multimodal mental health profiles [18,34,71,72,73].

The potential of this integration is profound. The stimulation parameters could be fine-tuned according to circadian rhythms, physical activity, affective states, or early warning signs of clinical relapse. It may also redefine the patient–clinician–device relationship, allowing for more seamless, intuitive, and responsive interaction across all levels of care [34,71,72,73].

Nevertheless, the implementation of these technologies raises substantial ethical and logistical considerations. Issues related to privacy, data security, patient autonomy, equitable access, and the necessity for robust clinical oversight must be addressed with care. Furthermore, it is important to ensure that the availability of such tools does not promote over-medicalization or foster unnecessary technological dependence.

**Figure 2:**
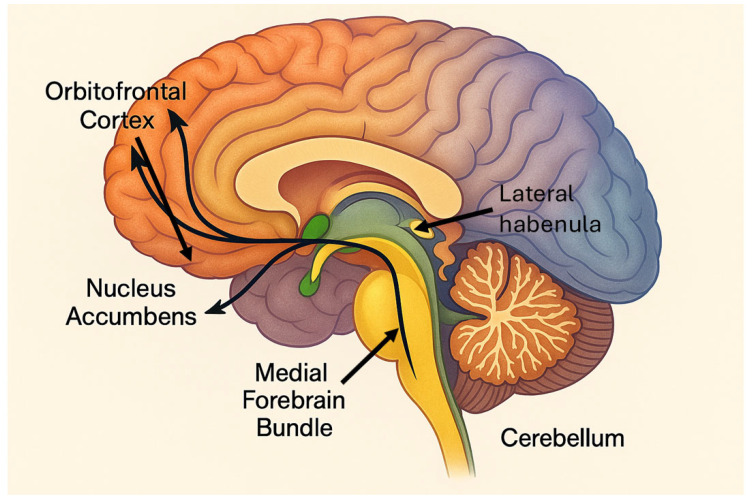
Schematic representation of emerging targets in psychiatric neurosurgery. The illustration highlights key structures within the limbic and reward-related circuitry, including the orbitofrontal cortex, nucleus accumbens, mid-forebrain bundle, and lateral habenula. These regions are currently under investigation as potential targets for DBS in treatment-resistant psychiatric disorders such as major depression and obsessive–compulsive disorder. This figure was created with the assistance of generative artificial intelligence for illustrative purposes only.

**Figure 3:**
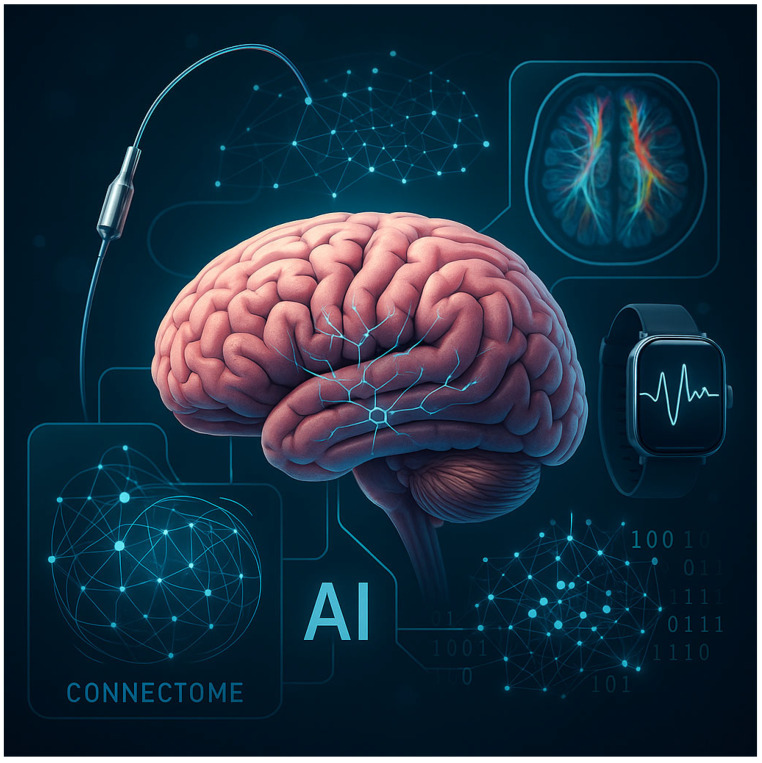
Conceptual representation of the future of psychiatric neurosurgery. The illustration depicts the integration of advanced neuromodulation technologies with real-time brain monitoring, wearable devices, and AI-driven analytics, reflecting a paradigm shift toward personalized and adaptive surgical interventions for severe psychiatric disorders. The image was created using generative artificial intelligence for illustrative purposes only.

Despite these concerns, the merging of neurosurgical treatment with wearable neurotechnology represents a tangible and promising direction for the field. It reimagines psychiatric intervention not as a static event, but as a dynamic and context-sensitive process—one that adapts continuously to the complexity of each patient’s lived experience (Figure 2 and Figure 3).

## 11. The Boundaries of Psychosurgery: Restoration or Enhancement?

Psychosurgery has historically been viewed as a last-resort therapeutic option for individuals with severe, treatment-resistant psychiatric disorders. However, with the rapid progress of neuroscience and brain mapping technologies, a provocative question has surfaced: Should psychosurgery remain confined to restoring impaired functions, or could it one day be used to enhance cognitive abilities such as memory, attention, or even intelligence?

The idea of selectively modulating specific neural circuits to boost high-level mental functions opens up intriguing possibilities, but it also raises serious ethical concerns. Techniques like DBS and transcranial magnetic stimulation (TMS) have occasionally produced unexpected side effects, influencing decision-making, motivation, or creativity. Although these outcomes are typically unintended, they demonstrate that cognitive enhancement via neurosurgical intervention is not purely hypothetical—it may be within reach.

Yet, the line between treatment and enhancement is increasingly blurred. Is it ethically justifiable to use invasive brain interventions to improve memory in otherwise healthy individuals? Should neuroenhancement be permitted in highly competitive environments such as academia or the workplace? What would the societal consequences be in terms of fairness, accessibility, and autonomy?

These concerns multiply when broader issues are considered, including the following:

- Social or professional pressure to undergo enhancement procedures;
- The risk of unintentional personality or identity changes;
- Unequal access to cutting-edge neurotechnologies;
- Potential coercive or military use of neuromodulation tools.

Given these risks, many experts advocate for strict regulation that limits the use of psychosurgery to clearly defined therapeutic purposes, postponing or prohibiting its application for enhancement in healthy individuals [72,74].

Ultimately, psychosurgery is at a crossroads: it could evolve into a precise medical tool aimed at restoring lost functions or it could usher in an era of neuroenhancement, raising complex ethical, legal, and societal questions that remain unresolved.

## 12. Conclusions

Psychiatric neurosurgery is entering a new era, shaped by technological innovation and a deeper understanding of brain networks. Techniques such as DBS, connectome-guided targeting, and adaptive closed-loop systems are redefining what is possible in the treatment of severe, treatment-resistant psychiatric disorders.

Although these interventions are still under clinical investigation, their rapid evolution suggests a future where the surgical modulation of dysfunctional circuits becomes increasingly precise, responsive, and tailored to individual neurobiological profiles. The integration of neural recordings, wearable sensors, and AI-driven decision-making offers a powerful framework for the continuous, personalized adjustment of therapeutic strategies.

In parallel, minimally invasive lesion-based approaches—such as focused ultrasound and stereotactic radiosurgery—are also gaining ground. These technologies provide alternatives for patients who are not candidates for implanted devices, and may offer durable effects with reduced procedural burden, particularly as imaging and targeting methods become more refined.

As neurosurgical psychiatry advances, key challenges remain: optimizing patient selection, validating long-term efficacy, managing ethical implications, and ensuring equitable access. But the direction is clear: psychiatric surgery is evolving from static anatomical intervention to the dynamic, data-informed modulation of the brain’s most complex circuits. Though not yet routine, these technologies mark a decisive step toward a more intelligent, connected, and individualized form of therapeutic intervention.

Table 1 provides a comparative overview of key technologies employed in psychiatric neurosurgery versus their use in neurological neurosurgery. While many of the core tools—such as DBS, lesioning techniques, and radiosurgery—are technically shared, their application in psychiatric disorders differs substantially in terms of target selection, circuit-level goals, and ethical implications. Psychiatric neurosurgery is uniquely focused on modulating neural circuits related to mood, emotion, and behavior, which introduces additional complexity regarding patient outcomes, identity, and consent.

## Figures and Tables

**Table 1:** Comparative applications of advanced neurosurgical technologies in psychiatric versus neurological disorders. This table summarizes the current and emerging uses of various neurosurgical techniques across psychiatric and neurological indications, highlighting differences in clinical maturity, targets, and integration of novel tools such as connectomics and artificial intelligence.

Technology/Technique	Use in Psychiatric Neurosurgery	Use in Neurological Neurosurgery
Deep Brain Stimulation (DBS)	In research for MDD, OCD, Tourette’s, and other psychiatric indications; modulates emotion/motivation circuits	Standard for Parkinson’s, essential tremor, and dystonia
Stereotactic Radiofrequency Lesioning	Used for OCD, MDD, and aggression; targets affective circuit nodes	Used in pain syndromes and movement disorders
Gamma Knife Radiosurgery	Used for non-invasive lesioning in OCD, depression, and aggressiveness	Used in arteriovenous malformations, trigeminal neuralgia, and tumors
High-Intensity Focused Ultrasound (HIFU)	MRI-guided capsulotomy for OCD; emerging use	Approved for tremor and pain
Irreversible Electroporation (IRE)	Experimental; used to target circuits with minimal damage	Under exploration for gliomas, epilepsy, and functional mapping
Closed-Loop (Adaptive) DBS	In research, adaptively modulates symptoms in MDD/OCD based on real-time signals	Being tested in Parkinson’s disease and epilepsy
Connectomic-Guided Targeting	Tailors targets based on individual connectivity profiles in OCD/MDD	Primarily used in movement disorders for targeting motor pathways
AI in Surgical Planning	Used to predict DBS outcomes and refine targeting in psychiatric indications	Emerging use in optimizing Parkinson’s DBS and seizure localization

## Data Availability

Not applicable.
